# Experimental Investigation on Mechanism of Latent Heat Reduction of Sodium Acetate Trihydrate Phase Change Materials

**DOI:** 10.3390/ma13030584

**Published:** 2020-01-26

**Authors:** Liu Wu, Jianqiang Li, Hui Wang, Ying Zhang, Shaowei Feng, Yongchang Guo, Jianling Zhao, Xixin Wang, Lijiang Guo

**Affiliations:** 1 School of Materials Science and Engineering, Hebei University of Technology, Tianjin 300130, China; 2 National Engineering Laboratory for Hydrometallurgical Cleaner Production Technology, Key Laboratory of Green Process and Engineering, Institute of Process Engineering, Chinese Academy of Sciences, Beijing 100190, China

**Keywords:** phase change materials, sodium acetate trihydrate, phase separation, latent heat reduction

## Abstract

Sodium acetate trihydrate (SAT) phase change material (PCM) has been well known for thermal energy storage due to its high latent heat and resource abundance. However, SAT suffers from severe latent heat reduction after heating and cooling cycles. Although a few of previous researches showed the reduction could be effectively inhibited by using thickeners, the mechanisms of the reduction process and thickeners’ inhibition have not been deeply explored till now. In this work, SAT modified by 5 wt.% nucleating agent of disodium hydrogen phosphate dodecahydrate (SAT/5 wt.% DSP) was prepared and 200 thermal cycles were carried out. The differential scanning calorimeter, Rheometer, X-ray diffractometry, and scanning electron microscope were used to investigate the extent of latent heat reduction, viscosity, phase composition and microstructure, respectively, and the infrared thermal imaging method was used to evaluate heat storage capacity. It was found that the latent heat of SAT/5 wt.% DSP dropped dramatically and the relative decrease in latent heat was measured to be 22.44%. The lower layer of SAT/5 wt.% DSP contained 24.1 wt.% CH_3_COONa, which was quantitatively consistent with the reduction extent. Furthermore, the phase change endothermic time of the lower layer was only 44.1% of that of the upper. SAT/5 wt.% DSP was further modified by 3 wt.% thickener of carboxymethyl cellulose (SAT/5 wt.% DSP/3 wt.% CMC) and endured 200 thermal cycles. The extent of the latent heat reduction of SAT/5 wt.% DSP/3 wt.% CMC was only 9.29%, and phase compositions were more homogeneous. The 3 wt.% CMC increased viscosity by 14 times, which effectively prevented the Stokes sedimentation velocity of CH_3_COONa in melts and inhibited the final macroscopic phase separation.

## 1. Introduction

Phase change materials (PCMs) for storing and releasing energy represent promising energy storage media to solve the mismatch between energy supply and demand [1,2,3,4,5,6]. In recent years, it has been widely used in the field of thermal energy storage, such as industrial waste heat recovery [7,8], building heating [9,10,11,12], as well as solar energy systems [13,14,15]. Inorganic salt hydrate PCMs are considered as promising candidates owing to their merits of high latent heat, non-flammability, constant phase change temperature, and low cost [16,17]. As a typical inorganic salt hydrate PCM, sodium acetate trihydrate (SAT, CH_3_COONa·3H_2_O) possesses high latent heat over 250 kJ/kg compared with other hydrated salt PCMs and a phase change temperature of 58 °C, which offers great potential applications in the field of thermal energy storage [18,19,20].

Unfortunately, SAT suffers from large supercooling, which results in unpredictable crystallization that prevents stored heat from being released at phase change temperature during the cooling process [21,22]. Hence, considerable efforts have been made to overcome this shortcoming in recent years. Hu et al. [23] used AlN (aluminium nitride) nanoparticles as nucleating agent of SAT. The supercooling degree of SAT could be reduced to 0–2.4 °C by adding 3–5 wt.% AlN nanoparticles. Mao et al. [24] investigated the effect of several nucleating agents on the supercooling of SAT. The results indicated that the minimum supercooling degree was 1.5 °C when the addition amount of disodium hydrogen phosphate dodecahydrate (DSP) was 6 wt.% in SAT. Similarly, silver nanoparticles [25], nano-copper [26] and iron oxide nanoparticles (α-Fe_2_O_3_) [27] were used as nucleating agents to reduce the supercooling of SAT by researchers. Among which, DSP is an ideal nucleating agent considering the cost of the large-scale practical application of SAT compared with nanoparticles nucleating agents. Moreover, severe phase separation still occurs in SAT when the supercooling is lower [28]. Cabeza et al. [29] showed that cellulose was the appropriate thickener compared with starch and bentonite. The effect of thickeners including super-absorbent polymers, polyvinyl alcohol and carboxymethyl cellulose (CMC) on SAT was reported by Ryu et al. [30]. The phase separation of SAT was successfully inhibited by thickening with CMC. Furthermore, Dannemand et al. [31] discovered that the latent heat reduction of SAT composite PCMs could be effectively inhibited by using CMC as a thickener, and latent heat was consistently around 205 kJ/kg over the six test cycles. However, thickeners (such as CMC) are used to inhibit the phase separation of hydrated salt PCMs, but the current thickeners still cannot fundamentally solve the latent heat reduction of hydrated salts. Solving the latent heat reduction of hydrated salt PCMs in practical applications is still a challenging research direction.

In this work, the latent heat reduction process of SAT was analyzed during 200 heating and cooling cycles. Importantly, the mechanism of the thickener inhibiting latent heat reduction was analyzed. A small amount of CMC increased the viscosity of solution, which prevented effectively the Stokes sedimentation velocity of CH_3_COONa in melts and inhibited the final macroscopic phase separation. This study provides theoretical guidance for latent heat reduction studies of SAT and other hydrated salts in practical applications.

## 2. Materials and Methods

### 2.1. Materials

SAT (CH_3_COONa·3H_2_O), DSP (Na_2_HPO_4_·12H_2_O) and CMC (carboxymethyl cellulose, analytical reagent grade, purity > 99%) were purchased from Sinopharm Chemical Reagent Co., Ltd. (Beijing, China).

### 2.2. Preparation of SAT Composite PCMs

CMC and DSP were used as thickener and nucleating agent to restrain phase separation and reduce supercooling, respectively. The composition of SAT/DSP/CMC (SAT modified by DSP and CMC) is listed in Table 1. It was reported that when the addition amount of CMC is 3 wt.%, the phase separation of SAT was effectively inhibited, and the phase change performance was optimal [24]. Every sample was thoroughly mixed by magnetic stirring in a 150-mL beaker in a water bath with a constant temperature of 80 °C, and then transferred to a 50 mL centrifuge tube with a cover, avoiding water evaporation.

### 2.3. Characterization

The viscosity (liquid state) was measured by Rheometer (DHR-2, TA Instruments, New Castle, DE, USA) at 80 °C. The latent heat was measured using the differential scanning calorimeter (DSC, Mettler-Toledo, Stockholm, Sweden), setting temperature from 25 to 90 °C with a heating/cooling rate of 10 °C/min under N_2_ at a flow rate of 50 mL/min. The component was analyzed by X-ray diffractometry (XRD, Smartlab, Rigaku Corporation, Tokyo, Japan). The morphology of the sample was observed by an environmental scanning electron microscope (ESEM, Quanta Feg 250, FEI Corporation, Hillsboro, OR, USA). The temperature of the sample was recorded using an infrared thermal camera (226, Fotric, Allen, TX, USA).

### 2.4. Supercooling Determination

The supercooling degree ΔT of the PCM can be calculated by the following formula [12]:

(1) $$\Delta T=T_{m}-T_{s}$$

where T_m_ is the melting temperature and T_s_ represents the solidification temperature. The purpose of this work is to study the reduction of latent heat of SAT during practical application, so DSP was used to reduce the supercooling of SAT. The cooling curve method was used to determine the supercooling degree of SAT composite PCMs. The as-prepared samples in the 50 mL centrifuge tubes were melted in a water bath with a constant temperature of 80 °C, and then cooled naturally at room temperature. Further, the temperatures of samples were recorded once a second by multichannel temperature recorder. Every sample was tested three times.

### 2.5. Heat Storage Capacity Determination

**Figure 1:**
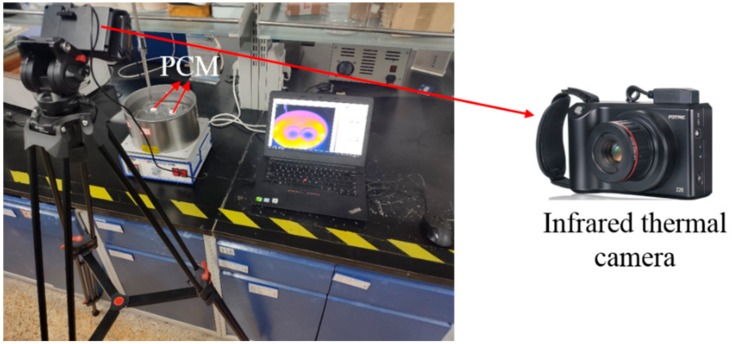
The equipment of heat storage capacity determination.

The heat storage capacity was qualitatively assessed by comparing the phase change time of samples under the same heating condition. Infrared thermal imaging method was used to observe the temperature changes of sample in heating and cooling process. The samples were pressed into wafers with the diameter of 2 cm and the quantity of 1 g, and then put in a glass dish without covering, floating above the oil bath of 80 °C. The temperatures of samples were recorded using an infrared thermal camera. The equipment is shown in Figure 1.

### 2.6. Thermal Stability Determination

The thermal stabilities of SAT/5 wt.% DSP and SAT/5 wt.% DSP/3 wt.% CMC were determined by comparing the latent heat changes before and after heating and cooling cycles. Four 1-g samples of SAT/5 wt.% DSP were placed in 1.5 mL centrifuge tubes. Then these centrifuge tubes were immersed in a water bath at the constant temperature of 80 °C. When the temperatures of samples didn’t change, they were quickly placed into the 20 °C water bath. Four samples were cycled 40, 80, 150, and 200 times as described above. Then every resulting sample was taken out of the centrifuge tubes, and ground uniformly, and then a few milligrams of powder sample were used for DSC testing. Same thermal stability test process of SAT/5 wt.% DSP/3 wt.% CMC was carried out. Every sample was tested five times. The error of the DSC test result is no more than 10 J/g.

## 3. Results and Discussion

### 3.1. Influence of DSP on Supercooling

**Figure 2:**
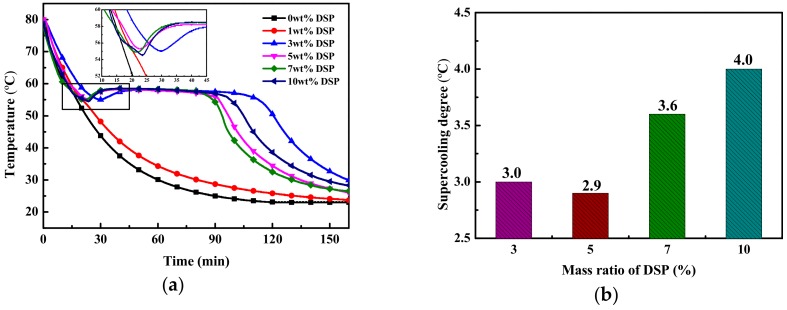
(**a**) Cooling curves and (**b**) supercooling degrees of SAT modified by different contents of DSP.

Cooling curves and supercooling degrees of SAT modified by different contents of DSP are illustrated in Figure 2. It can be observed that the SAT/1 wt.% DSP (SAT with 1 wt.% DSP) doesn’t solidify, while the SAT with 3 wt.% to 10 wt.% DSP solidified with some extent of supercooling. When the content of DSP is 5 wt.%, the supercooling degree is 2.9 °C, which is the lowest as shown in Figure 2b.

### 3.2. The Analysis of Latent Heat Reduction

**Figure 3:**
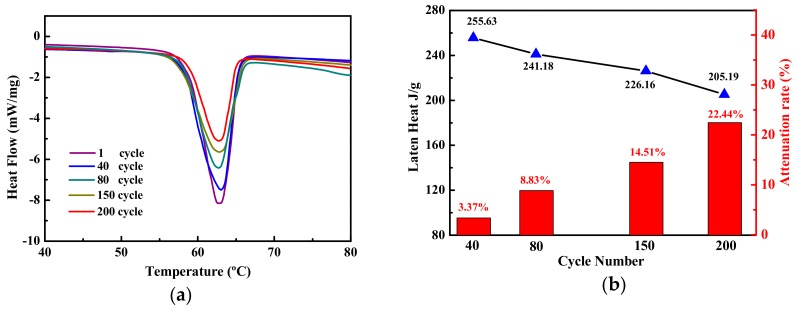
(**a**) DSC melting curves, (**b**) latent heat change curve and reduction extent of SAT/5 wt.% DSP at different cycles.

The 200 heating and cooling cycles of SAT/5 wt.% DSP were carried out. The DSC measurements of SAT/5 wt.% DSP in different cycles are shown in Figure 3, and a concave peak is observed in each DSC melting curve. As the number of cycles increases, the area of the peak decreases. This indicates that the latent heat of SAT/5 wt.% DSP is decreasing during 200 heating and cooling cycles. The extent of latent heat reduction was calculated by the following formula [32]:

(2) $$\alpha =\frac{\left(\Delta H_{1}-\Delta H_{2}\right)}{\Delta H_{1}}\times 100\%$$

where α is the extent of latent heat reduction, ΔH_1_ represents the latent heat of the initial sample, and ΔH_2_ is the latent heat of sample after different thermal cycles.

The five DSC test results of the SAT/5 wt.% DSP are shown in Table 2. The experimental results are the mean values of five test results. The standard deviations of the latent heat for 40, 80, 150, 200 cycles are calculated respectively as 4.72, 5.03, 4.58, 4.52. After 200 heating and cooling cycles, the latent heat of SAT/5 wt.% DSP drops to 205.19 J/g as shown in Figure 3b. The extent of latent heat reduction of SAT/5 wt.% DSP increases from 3.37% in 40 cycles to 22.44% in 200 cycles.

**Figure 4:**
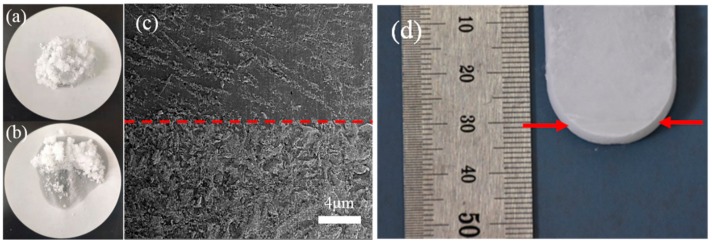
(**a**) The original sample of SAT/5wt%DSP and (**b**) the sample cycled 200 times; (**c**) SEM image of lower sample of SAT/5wt%DSP; (**d**) the profile of SAT/5wt%DSP.

Figure 4a is the 52.5g original sample of SAT/5 wt.% DSP and Figure 4b shows the sample cycled 200 times. The uncrystallized liquid phase was observed in SAT/5 wt.% DSP after 200 heating and cooling cycles in Figure 4b. The 200 cycled sample in the 50 mL centrifuge tube was cut vertically from the top to the bottom of the centrifuge tube, and the profile is shown in Figure 4d. The morphology at a bottom height of 3 cm of SAT/5 wt.% DSP is illustrated in Figure 4c. Different morphologies are observed on both sides of the interface, which indicates that the components on both sides of the interface are different. It should be pointed out that phase separation occurs in SAT/5 wt.% DSP after 200 heating and cooling cycles.

**Figure 5:**
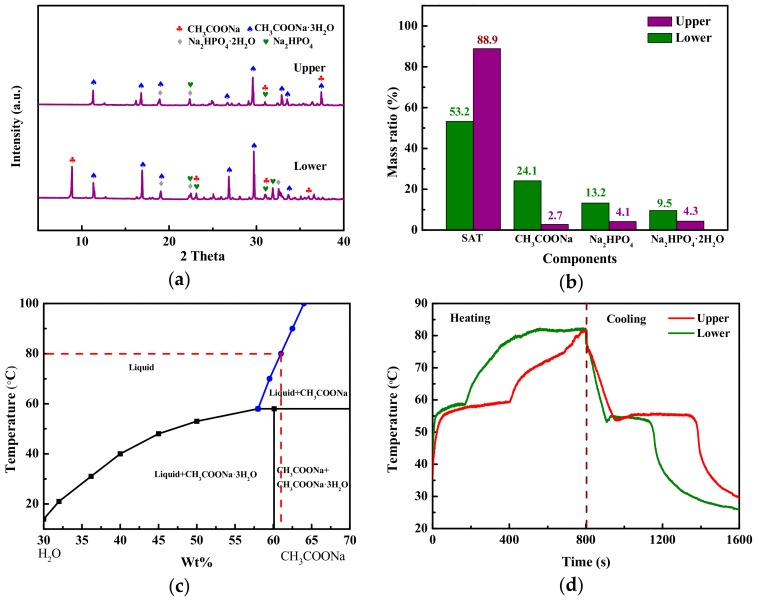
(**a**) XRD patterns and (**b**) semi-quantitative analyses of upper and lower samples of SAT/5 wt.% DSP; (**c**) Phase diagram of sodium acetate-water system [13]; (**d**) Temperature–time curves of upper and lower samples of SAT/5 wt.% DSP.

Furthermore, the SAT/5 wt.% DSP was cut horizontally at the interface and divided into two layers: the upper and lower samples. The crystalline phases of upper and lower samples were analyzed by XRD as shown in Figure 5a. SAT exhibits strong peaks at 11.3°, 16.8°, and 29.58° (2 Theta) observing in the XRD pattern of the upper sample. Furthermore, Na_2_HPO_4_·2H_2_O and Na_2_HPO_4_ show weak peaks, which is caused by the decomposition of DSP [33,34]. The CH_3_COONa is observed at 8.83° and 23.08° in the XRD pattern of the lower sample, which confirms that phase separation has occurred in SAT/5 wt.% DSP. Figure 5b shows the semi-quantitative analysis results of the upper and lower samples by k value method. It can be seen from the Figure 5b that the component of lower sample is mainly SAT with a mass ratio of 53.2%, and the 24.1 wt.% of CH_3_COONa of lower sample is much higher than 2.7 wt.% of the upper sample. As presented in Figure 5c, SAT melts to form saturated sodium acetate solution at 80 °C [13]. The saturated sodium acetate solution and CH_3_COONa have densities of 1280 kg/m^3^ and 1517 kg/m^3^ [13]. Therefore, CH_3_COONa sinks to the bottom owing to higher density when SAT/5 wt.% DSP is in a molten state, leading to aggregate at the lower layer and the formation of an interface between the sediment and liquid phase.

**Figure 6:**
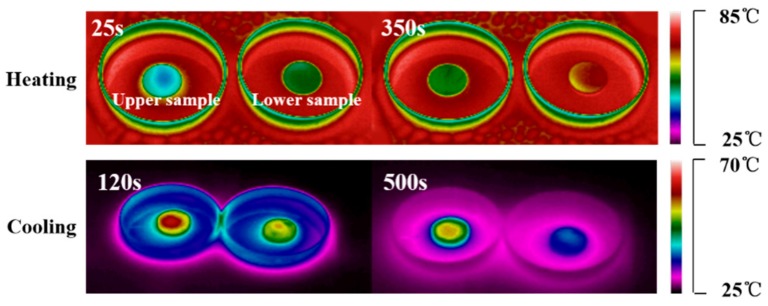
Infrared ray (IR) images of upper and lower samples of SAT/5 wt.% DSP during heating and cooling process.

The temperature–time curves (Figure 5d) and temperature-distribution images (Figure 6) of upper and lower samples of SAT/5 wt.% DSP were obtained using an infrared thermal camera. As shown in Figure 5d, the temperature-time curves of the upper and lower samples have two temperature plateaus around 55–58 °C, which represent thermal energy storage and release in the form of latent heat. The phase change endothermic time of the lower sample is only 150s, but 340s of that for the upper sample. The shorter phase change endothermic time of the lower sample is due to the fact that it only contains 53.2 wt.% SAT. It is worth noting that the phase change of lower sample firstly occurs when the heating time is 25s, because its sensible heat is lower than that of the upper sample. When the heating time is 350s, the temperature of the upper sample is 58 °C indicating that the phase change is still under way, while the lower sample has ended the phase change process and its temperature reaches to 77 °C. It can be inferred that the latent heat of SAT/5 wt.% DSP decreases by 22.44%, which is because 24.1 wt.% CH_3_COONa without heat storage capacity aggregates at the lower layer.

### 3.3. The Analysis of Inhibiting Latent Heat Reduction

**Figure 7:**
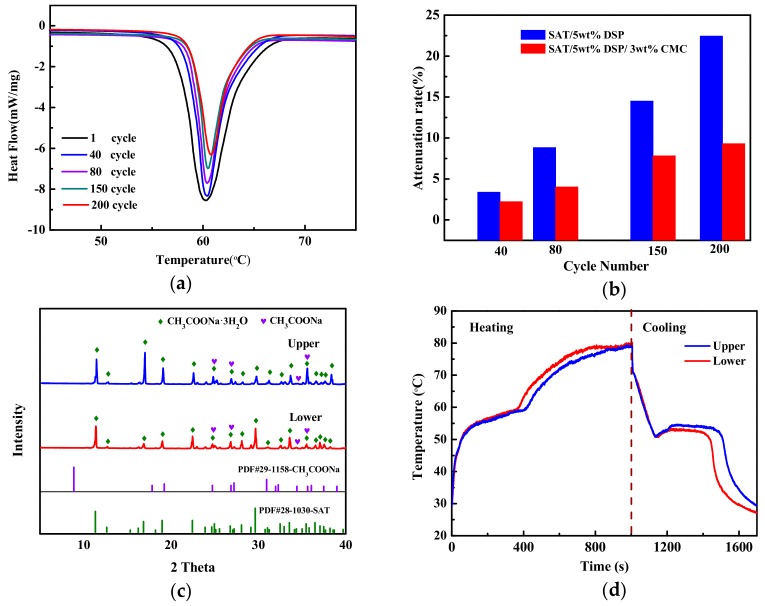
(**a**) DSC melting curves of SAT/5 wt.% DSP/3 wt.% CMC and (**b**) the extent of latent heat reduction of SAT/5 wt.% DSP and SAT/5 wt.% DSP/3 wt.% CMC at different cycles; (**c**) XRD patterns and (**d**) temperature–time curves of upper and lower samples of SAT/5 wt.% DSP/3 wt.% CMC.

The DSC measurements of SAT/5 wt.% DSP/3 wt.% CMC during different heating and cooling cycles was shown in Figure 7a. The areas of concave peak decrease with the increase of the cycle number of SAT/5 wt.% DSP/3 wt.% CMC. The extent of latent heat reduction of SAT/5 wt.% DSP/3 wt.% CMC was calculated by formula (2) as presented in Figure 7b. The 3 wt.% CMC reduces the extent of latent heat reduction of SAT/5 wt.% DSP from 22.44% to 9.29%. The SAT/5 wt.% DSP/3 wt.% CMC was divided into two layers (the upper and lower samples) in the same way as that of SAT/5 wt.% DSP. Figure 7c represents the XRD patterns of upper and lower sample of SAT/5 wt.% DSP/3 wt.% CMC, and the characteristic peaks of SAT at 11.3°, 16.8°, and 29.58° (2 Theta) are observed in the XRD patterns of the upper and lower samples. The CH_3_COONa shows weak peaks at 24.73°, 26.86°, 34.41°, and 35.62° (2 Theta) in the XRD patterns of the upper and lower samples. The results indicate that there is slight phase separation in SAT/5 wt.% DSP/3 wt.% CMC.

**Figure 8:**
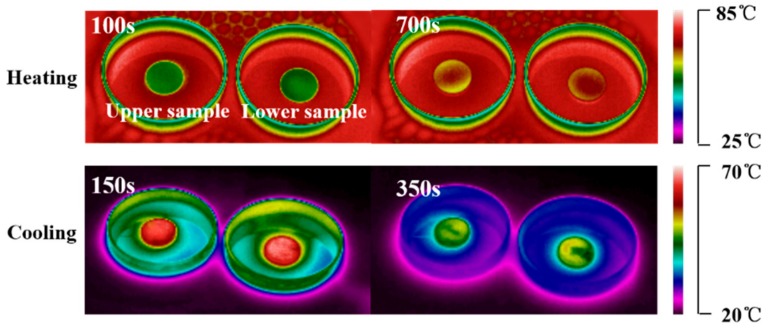
IR images of upper and lower samples of SAT/5 wt.% DSP/3 wt.% CMC during heating and cooling process.

The temperature–time curves and temperature-distribution images of upper and lower samples of SAT/5 wt.% DSP/3 wt.% CMC are shown in Figure 7d and Figure 8, respectively. As illustrated in Figure 7d, there are two temperature plateaus with only slight changes on the temperature–time curves of upper and lower samples of SAT/5 wt.% DSP/3 wt.% CMC. This indicates that the phase change endothermic time of the lower sample of SAT/5 wt.% DSP/3 wt.% CMC is consistent with that of the upper, which is attributed to the uniform composition of the upper and lower samples. It can be concluded that 3 wt.% CMC can effectively slow down the extent of latent heat reduction of SAT by inhibiting the phase separation.

**Figure 9:**
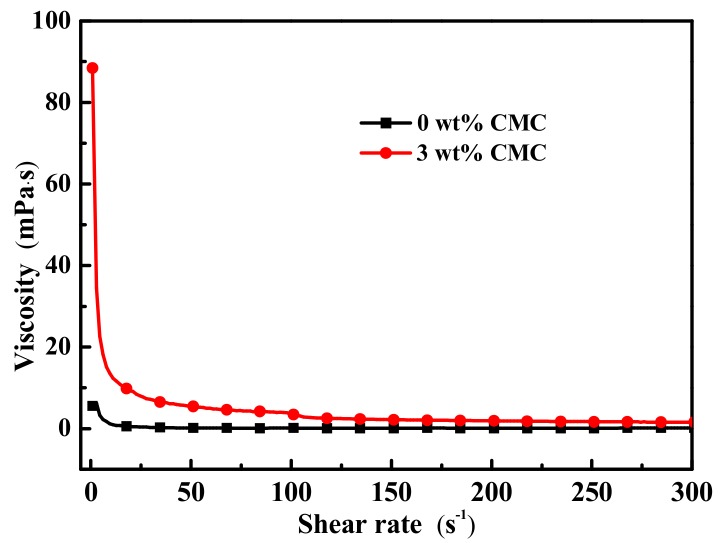
Viscosity curves of SAT/5 wt.% DSP and SAT/5 wt.% DSP/3 wt.% CMC at 80°C.

To further investigate the mechanism of thickeners’ inhibition, the viscosity of the sample was measured. The viscosity curves of SAT/5 wt.% DSP and SAT/5 wt.% DSP/3 wt.% CMC after 200 heating and cooling cycles at 80 °C are shown in Figure 9. The viscosities of SAT/5 wt.% DSP and SAT/5 wt.% DSP/3 wt.% CMC are 6.02 mPa·s and 88.50 mPa·s, respectively. The relationship between the Stokes settling velocity of anhydrous salt and viscosity of solution can be evaluated by Stokes law [35]:

(3) $$V=\frac{D\left(\rho_{p}-\rho_{b}\right)r^{2}}{\eta }$$

where V is the Stokes settling velocity of anhydrous salt, D is constant, ρ_p_ and ρ_b_ represent anhydrous salt density and solution density, r is radius of anhydrous salt, and η is the viscosity of solution.

According to the Stokes law, the Stokes settling velocity of anhydrous salt is inversely proportional to the viscosity of solution. Provided that r of CH_3_COONa in SAT/5 wt.% DSP and SAT/5 wt.% DSP/3 wt.% CMC does not change. The Stokes settling velocity of CH_3_COONa in SAT/5 wt.% DSP/3 wt.% CMC is 0.0027r^2^ m/s, which is 8.07 times lower than 0.0218r^2^ m/s of SAT/5 wt.% DSP. During the heating process of SAT/5 wt.% DSP, CH_3_COONa rapidly settles to the bottom on account of low viscosity and forms a solid-liquid interface, which prevents the combination of CH_3_COONa below the interface with the liquid phase in cooling process. When SAT/5 wt.% DSP is cooling, the CH_3_COONa under the interface does not undergo phase change. During certain heating and cooling cycles, as the heating and cooling cycle increases, more and more CH_3_COONa without heat storage capacity will settle down, resulting in the reduction of latent heat. Inversely, the settling velocity of CH_3_COONa is significantly reduced due to the higher viscosity during the heating process of SAT/5 wt.% DSP/3 wt.% CMC, so that a large proportion of CH_3_COONa can’t aggregate at the lower layer and continue to phase change in cooling process. Thus, its latent heat is relative reduced.

## 4. Conclusions

The mechanisms of SAT latent heat reduction process and the thickeners’ inhibition were investigated. SAT modified by 5 wt.% disodium hydrogen phosphate dodecahydrate (SAT/5 wt.% DSP) and further modified by 3 wt.% carboxymethyl cellulose (SAT/5 wt.% DSP/3 wt.% CMC) were prepared and 200 heating and cooling cycles were carried out. For the SAT/5 wt.% DSP, the results showed that 24.1 wt.% CH_3_COONa aggregated at the lower layer of SAT/5 wt.% DSP, forming a solid-liquid interface and preventing the phase change of CH_3_COONa under the interface during the cooling process. As a result, the latent heat of SAT/5 wt.% DSP decreased with the increase of the heating and cooling cycles. In contrast, the phase composition of SAT/5 wt.% DSP/3 wt.% CMC was more homogeneous. The viscosity of SAT/5 wt.% DSP/3 wt.% CMC was 14 times higher than that of SAT/5 wt.% DSP, and the settling velocity of CH_3_COONa in SAT/5 wt.% DSP/3 wt.% CMC reduced by 8.07 times. Therefore, a large proportion of CH_3_COONa interacted with liquid phase during cooling process, which slow down the extent of latent heat reduction.

## Figures and Tables

**Table 1:** Samples composition of SAT/DSP/CMC.

Samples	SAT (g)	DSP (g)	CMC (g)	The mass ratio of SAT:DSP:CMC
1	50	0.5	0	1:0.01:0
2	50	1.5	0	1:0.03:0
3	50	2.5	0	1:0.05:0
4	50	3.5	0	1:0.07:0
5	50	5.0	0	1:0.1:0
6	50	2.5	1.5	1:0.05:0.03

**Table 2:** The five DSC test results of the SAT/5 wt.% DSP.

Test Frequency	40 Cycle (J/g)	80 Cycle (J/g)	150 Cycle (J/g)	200 Cycle (J/g)
1	251.84	236.15	220.73	199.65
2	253.67	248.23	233.59	202.37
3	262.98	239.82	222.64	203.28
4	259.15	245.83	228.76	208.43
5	250.51	235.87	225.08	212.22
